# An Analysis of Different Techniques Used to Seal Post-Extractive Sites—A Preliminary Report

**DOI:** 10.3390/dj10100189

**Published:** 2022-10-09

**Authors:** Paolo Pesce, Eitan Mijiritsky, Luigi Canullo, Maria Menini, Vito Carlo Alberto Caponio, Andrea Grassi, Luca Gobbato, Domenico Baldi

**Affiliations:** 1 Department of Surgical Sciences, University of Genova, 16100 Genoa, Italy; 2 Department of Otolaryngology, Head and Neck and Maxillofacial Surgery, Tel-Aviv Sourasky Medical Center, Sackler Faculty of Medicine, Tel-Aviv University, Tel Aviv 6139001, Israel; 3 The Maurice and Gabriela Goldschleger School of Dental Medicine, Tel Aviv University, Tel Aviv 6997801, Israel; 4 Department of Clinical and Experimental Medicine, University of Foggia, 71122 Foggia, Italy; 5 Private Practice, 42100 Reggio Emilia, Italy; 6 Private Practice, 35027 Padua, Italy

**Keywords:** alveolar ridge preservation, graft material, tooth extraction, socket preservation

## Abstract

Background: Bone grafting in post-extractive site improves tissue regeneration. Soft tissue sealing of the grafted post-extractive alveolus is supposed to limit microbiological contamination from the oral cavity and to stabilize the coagulum. Several techniques are presented in the literature to reach this goal using different heterologous matrices or autogenous grafts. In addition, recently, a technique based on the use of granulation tissue in the post-extractive alveolus has been proposed. Aim: To compare the effect of different graft sealing approaches in post-extractive sites by qualitatively evaluating their healing process. Materials and Methods: This retrospective investigation included 30 patients requiring post-extractive site regeneration in the aesthetic area. Post-extractive sites were regenerated using a bovine bone matrix and patients were divided into three groups (10 patients in each group) according to the material used to seal the alveolar socket. In the UD group, the granulation tissue was used to seal the defect; in the PC group, epithelial-connective soft tissue graft was used, and in the COLL group, a collagen-based membrane was employed. Images of the post-extractive sites at different follow-up periods (2 and 12 weeks) were taken and the healing process was blindly evaluated by two independent practitioners. The Healing Index (HI) by Landry, Turnbull and Howley was used to assess the quality of the healing process. The combination of presence/absence of five clinical criteria defines an HI ranging from 1 (very poor) to 5 (excellent). Patients’ clinical-pathological variables were recorded. One-way ANOVA was used to explore the dependence of HI on the different socket preservation protocols. Results: Based on clinical-pathological characteristics of the included patients, there were no statistically significant differences among the different sealing techniques. At the 2-week follow-up appointment, HI did not differ among the socket preservation protocols evaluated. Moreover, smoking status and reason for extraction did not influence the HI among the three groups (two-way ANOVA *p*-value = 0.686, *p*-value = 0.248 respectively). At the 2-week follow-up appointment, HI was significantly different among the socket preservation protocols investigated. Specifically, the group undergoing collagen-based socket preservation procedure reported the highest HI, compared to the other two techniques (COLL mean 4.60 ± 0.5; PC mean 3.5 ± 1.2; UD mean 3.4 ± 0.5, one-way ANOVA *p*-value 0.006). Conclusions: The use of collagen porcine membranes may represent a suitable option to improve the patient healing process in grafted post-extractive sites together with reducing the surgical intervention time compared to alternative sealing techniques.

## 1. Introduction

Numerous animal and clinical studies have demonstrated that undisturbed wound healing following tooth extraction leads to loss of the alveolar ridge volume and change in ridge shape [1,2,3,4]. It has been shown that up to 50% of the horizontal bone can be lost within 12 months’ post-extraction [1]. At the same time, the increasing esthetic demands of patients challenges clinicians, especially when an implant-supported rehabilitation of the premaxilla is needed [5,6]; implant location should comply with patient esthetical, functional, and phonetical needs [7]. To satisfy these requests, clinically healthy and volumetrically adequate bone and soft tissues are needed for a prosthetically-driven implant position [8], which are essential preconditions for the success and long-term maintenance of the implant-supported restoration [9,10].

To maintain adequate bone and soft tissue levels, different ridge preservation and reconstruction techniques have been proposed [11,12,13,14,15].

The aim of these techniques is to reduce the shrinkage of hard and soft tissues during alveolar healing [16,17,18].

Bone grafting was proven to promote regeneration, with improved results when a membrane-limiting microbiological contamination from the oral cavity was used [19,20]. To reach this clinical goal, different materials were adopted, such as resorbable collagen-based matrices [2,21] and non-resorbable ePTFE matrices [22,23]. Nowadays, a commonly applied alveolar ridge preservation technique involves a flapless reconstruction with exposed membrane and secondary wound healing [24,25]. This technique safeguards minimal invasivity, mucogingival line preservation, and gain of keratinized soft tissue [26,27]. On the other hand, lack of sealing, because of degradation of resorbable materials [28] or the miss-adaptation of non-resorbable ones, could lead to clinical failure [29]. To overcome these disadvantages and improve clinical outcomes, a good management of soft tissue is mandatory [30,31,32,33]. Epithelial-connective soft tissue grafts picked up from the palate may be clinically effective, resulting in an aesthetic improvement with minimal post-operative discomfort after surgery [34,35]. More recently, granulation tissue used in post-extractive alveolus to seal the socket has been proposed and exhibited a good sealing, preventing oral contamination and achieving successful soft tissue healing [36,37].

So far, a consensus on the better sealing material is missing in the literature.

The aim of the present retrospective study was to compare the effect of different graft-sealing approaches in post-extractive sites by qualitatively evaluating their healing process. The null hypothesis was that no difference is present between the three different sealing techniques analyzed.

## 2. Materials and Methods

This retrospective study was conducted according to the Good Clinical Practice Guidelines (GCPs) and following the principle of the Declaration of Helsinki–ethical principles. Patients signed an informed consent. The current retrospective investigation included patients from three different private clinics (Rome, Padua, Milan). The study was approved by the ethical committee of the University of Genova (2021/44).

A convenient sample of 30 consecutive patients underwent extraction of a non-restorable anterior tooth, leading to a class 1 post-extractive defects with no buccal bone wall deficiency regenerated for implant placement purposes [38]. Each treatment was performed in a different clinic. Patients had to be at least 18 years old, in general good health conditions (ASA 1-2), and clinical images of the post-extractive site at different follow-up periods (2 and 12 weeks) were required to be present. A skilled clinician, different from the surgeons (V.C.A.C), retrospectively collected clinical data of the patients.

All patients received oral hygiene instructions and debridement 2 weeks before surgery. Prior to intervention, patients had to rinse with 0.2% chlorhexidine mouthwash for 1 min (Curasept, Curaden Healthcare, Saronno, Italy). All patients received prophylactic antibiotic therapy (amoxicillin and clavulanic acid 1 g, 1 h before tooth extraction and 1 g three times/day for the next 4 days) [39,40]. Treatment was performed in local anesthesia by articaine hydrochloride with epinephrine 1:100,000 (Orabloc, Pierrel, Milan, Italy). Teeth were extracted using a minimally invasive flapless approach followed by a soft tissue curettage and the post-extraction alveolus was debrided using an ultrasound tip (S2, W&H, Bürmoos, Austria). Bovine collagen–hydroxyapatite biomaterial (Bio-Oss Collagen, Geistlich, Wolhusen, Switzerland) was mixed with patients’ blood and inserted into the socket up to 3 mm from the gingival margin.

Patients were divided into three groups according to the material used to seal the post-extractive socket. First group underwent upside-down technique (UD) where the granulation tissue was used to seal the defect together with a collagen-based membrane (Bio-Gide, Geistlich Biomaterials, Wolhusen, Switzerland); in the second group, epithelial-connective soft tissue graft was used (PC) [41]. The latter included patients treated using a collagen-based membrane (COLL) (Bio-Gide, Geistlich Biomaterials, Wolhusen, Switzerland) [42]. In UD patients, the whole procedure was based on the salvage of the granulation tissue, which usually constitutes the consequence of the pathological process affecting teeth [43,44]. In these patients, granulation tissue was carefully detached from the surrounding alveolar bone. Over the graft material, a collagen-based membrane (Bio-Gide, Geistlich Biomaterials, Wolhusen, Switzerland) was used to seal the socket and the previously salvaged granulation tissue was used to cover the membrane by suturing.

In PC group, an epithelial-connective tissue graft was collected from the palate and sutured on the post-extractive site [41]. In COLL, a resorbable porcine-derived membrane (Bio-Gide, Geistlich Biomaterials, Wolhusen, Switzerland) was used as a sealer and kept in-site by suturing [45].

Patients were instructed to keep oral hygiene, limiting to soft brushing for the first 2 weeks around the surgical site and rinsing twice a day with 0.12% chlorhexidine [46].

Patients were recalled at 2 and 12 weeks after surgery, visited, and clinical pictures were collected.

Clinical information and images were extracted from patients’ files. Healing Index (HI) by Landry, Turnbull and Howley was used to assess the quality of healing process [25,47]. The combination of presence/absence of five clinical criteria (tissue color, response to palpation, granulation tissue, incision margin, suppuration) defines a HI ranging from 1 (very poor) to 5 (excellent).

**Figure 1:**
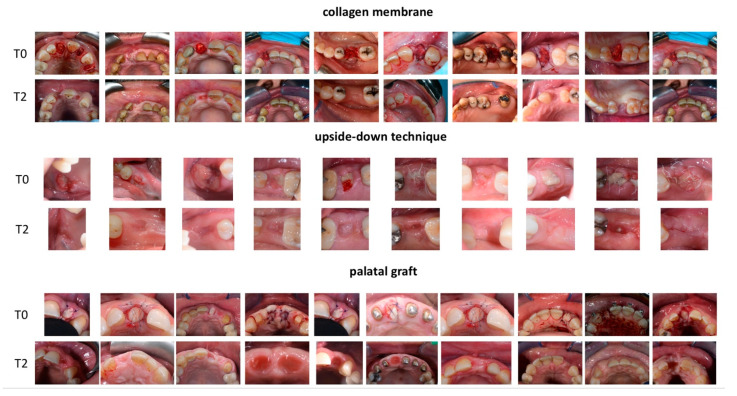
First group (collagen membrane) included patients were a collagen-based membrane was employed. In the second group (upside-down technique) a granulation tissue was used to seal the defect together with a collagen-based membrane. In the latter group, a palatal graft was used in order to seal the socket.

Two evaluators (V.C.A.C., E.M.) blinded to the surgical procedure independently provided an HI for each post-extractive site (Figure 1).

A third evaluator (L.C.) calculated a value of the k-statistic to ascertain the level of reviewers’ agreement. This last author also took a final decision of HI scoring after discussion with the first two reviewers in a joint meeting. Post-operative complications and adverse events were noted in the clinical record.

### Statistical Analysis

Patients’ clinical-pathological variables were recorded and differences among groups were investigated by chi-square test. The Healing Index was the main outcome and considered as a continuous variable. Normal distribution was explored through Shapiro–Wilk test [48]. Because of the non-normal distribution of the HI, non-parametric tests were furtherly performed, the Spearman rank test was used to investigate correlations among continuous variables, while the Mann–Whitney and Kruskal–Wallis tests were useful to investigate differences in means among groups. One-way ANOVA was used to explore the dependence of HI on the different socket preservation protocols, while two-way ANOVA was employed to investigate furtherly dependence of HI on different clinic-pathological variables.

## 3. Results

The final study analysis included 30 patients, which successfully completed the follow-up period check at 2 and 12 weeks. Each group (UD, PC, and COLL) included 10 patients. Quantity agreement with kappa showed strong agreement between evaluators [49] with a value of 0.853, such as 88.33% of observed agreements [50].

All patients highlighted no signs of suppuration or bleeding at the palpation.

Among the different socket preservation protocols, there were no differences in age, sex, reasons for extraction, and smoking status. Moreover, Mann–Whitney and Kruskal–Wallis tests showed no differences between HI and sex, reason for extraction, and smoking status. At last, the Spearman rank correlation test showed no correlation between HI and both age or time of surgery.

Clinical-pathological characteristics of included patients are reported in Table 1.

Patients undergoing PC protocol reported longest surgery time (80 ± 21 min) compared to both COLL protocol (38 ± 5 min, *p*-value < 0.001) and UD protocol (52 ± 23 min, *p*-value = 0.001).

At the 2 weeks’ follow-up check, HI did not differ among socket preservation protocols (COLL mean 1.90 ± 1; PC mean 1.90 ± 0.3; UD mean 2.00 ± 1.2, one-way ANOVA *p*-value 0.963). Moreover, smoking status and reason for extraction did not influence the HI among the three group protocols (respectively, two-way ANOVA *p*-value = 0.686, *p*-value = 0.248).

At the 12 weeks’ follow-up check, HI differed significantly among socket preservation protocols. Specifically, COLL group reported highest HI, compared to both PD and UD. (COLL mean 4.60 ± 0.5; PC mean 3.5 ± 1.2; UD mean 3.4 ± 0.5, one-way ANOVA *p*-value 0.006). Healing index at different follow-up times are summarized in Table 2. Reason for teeth extraction and smoking status did not influence healing index (two-way ANOVA *p*-value, respectively 0.108; 0.778).

## 4. Discussion

In post-extractive socket preservation, the use of a resorbable porcine-derived membrane to seal the xenograft regenerative material reported the best clinical healing outcome. In clinical practice, both soft and hard tissue preservation in post-extractive sites, are mandatory to satisfy patient esthetical, functional, and phonetical needs and guarantee the long-term success of the rehabilitation [7,10].

Results from this study showed that flapless reconstruction with biomaterials and secondary wound healing in sockets without soft tissue deficiency is a predictable technique, leading to a successful healing of the post-extraction site. In this complex healing process, close graft/bone contact, blood clot stability and sealing of the post-extractive site represent essential requirements to obtain the clinical success [51,52,53]. Recently, a systematic review and meta-analysis investigated the effects of different graft materials [54] and platelet concentrates [55,56], while limitations have been found on scientific evidence of sealing techniques in post-extractive sites [57]. Faria-Almeda et al., in a systematic review, stated that there is no consensus in using a soft tissue graft in alveolar preservation techniques, while few studies have compared alveolar preservation techniques with and without membrane, demonstrating that the application of a membrane allows improving alveolar ridge preservation [57]. Similar results were obtained in a more recently published systematic review and meta-analysis of Del Fabbro et al. [58], showing superior results in socket preservation associated to membrane sealing [57]. These results might be a consequence of the impact of isolating the socket environment from the oral cavity contaminants, above all bacteria [19]. Early stages of graft incorporation are led by the organization of a fibrin network evolving in granulation tissue [59]. However, the healing process represents a more complicated series of events and processes that include vascular alterations and inflammatory activation, letting migration, proliferation, and differentiation of distinct cell populations. An extracellular matrix is then produced leading to bone formation, modeling and remodeling, ending in the completion of the healing process [60]. Socket or ridge preservation consists in the allocation of graft material in the post-extractive site. Then, this material might be covered by a membrane or a rotated flap [61]. While the present technique promotes the biological processes described above, clinically the aim is to maximize the quantity of bone formation, limiting alterations of the ridge profile as a consequence of tooth extraction [62]. Traditionally, flap surgery has shown poor outcomes due to flap mobility which disturbs clot arrangement interfering with the correct healing process [63]. Nowadays, the use of flapless or flapped surgery in socket preservation is still controversial [64,65,66]. The outcomes of our study support the evidence by a Barone et al. study in which better preservation of keratinized mucosa and improved patients’ compliance in oral hygiene and aesthetic outcome was found when a collagen membrane sealing was used [64]. In our study, patients undergoing collagen membrane sealing benefitted of better wound healing in the 4 weeks of follow-up. Our results are in agreement with current literature evidence, showing that extraction sockets sealed by collagen membrane present significantly lower bone loss, compared to spontaneous healing [42]. Such improvements reflect also results from Carmagnola et al., where patients undergoing collagen membrane sealing showed higher quantity of lamellar and woven bone compared to grafting material-treated sockets [67]. These results might be consequence of the role of the membrane in preventing epithelial migration into bone defects, while preserving dimensional organization of the post-extractive site [68]. Favorable effects of collagen-based membranes are consequence of its dense surface, which prevents the bone defects to be filled of fibrous tissue, while stimulating bone-forming cells [69]. Moreover, collagen has many physicochemical properties, with hemostatic activity, chemotactic effects over gingival fibroblasts and permeability that allows toxin/nutrient exchange [70]. Based on previous considerations, membranes, and in particular collagen made membranes, are useful tool in blood clot stabilization, bone regeneration by keeping space in the socket, and protecting the post-extractive socket from mechanical disruption and oral contamination [53]. Our results also suggest that collagen-based membranes might reach improved clinical outcomes by additionally promoting wound healing, reducing patients’ discomfort and favoring oral hygiene, confirming data reported by Meloni et al. [34].

On the other hand, the healing process in the “upside technique” resulted completely differently. Teeth affected by deep caries or periodontal disease are often surrounded by inflammatory tissue [36]. This tissue is mainly made of chronic inflammatory cells and epithelium with low percentage of connective tissue [37] that can be used as sealing material of the post-extractive site using an immediate flapless technique. As demonstrated in the present study, once exposed to the oral environment, this tissue is able to protect and seal the graft. At the same time, this granulation tissue, once detached from the bony walls, tends to get transformed into epithelium. The clinical observation of soft tissue healing alone, without analyzing the effect of the different techniques on underlying bone regeneration and tridimensional volumetric changes is the main limitation of the present study. Another limitation is the retrospective design of the present investigation. Additionally, in both the COLL and UD groups, a collagen matrix was used reducing the treatment effect. However, a collagen matrix was used below the granulation tissue was to prevent its ingrowth into the graft material. The experimental rational behind this approach was to test if the collagen matrix itself has the same potentiality in soft tissue regeneration compared to the granulation tissue.

Our results suggested that smoking and reasons of extraction had no relation to the Healing Index; however, it must be pointed out that the number smoked cigarettes and the type of smoking (traditional vs. electronic) was not registered, and this could have influenced the results [71]. Dealing with the reason of extraction, it must be underlined that all patients assumed an antibiotic therapy that could have influenced the results.

This is the first study comparing traditional approaches (autologous soft tissue graft and collagen membrane) versus a promising new technique (granulation tissue elevation). However, some limits must be underlined. This is a retrospective pilot analysis with a small sample size and each technique was performed by a different operator. This may have affected the results.

Longer follow-up periods and randomized controlled trials may contribute in future studies to further assess the quality of the healing process.

## 5. Conclusions

Within its limitations, the study suggests that the use of collagen porcine membranes may represent a suitable option to improve patients’ healing process, together with reducing the surgical intervention time.

However, further studies are needed to confirm the better efficacy on bone preservation of this procedure when compared to surgical intervention requiring a connective graft.

## Figures and Tables

**Table 1:** Clinic-pathological characteristics of patients included in the study.

Clinic-Pathological Characteristics		COLL (tot. 10)	PC (tot. 10)	UD (tot. 10)	*p*-Value
Mean Age ± S.D.		51.80 ± 8	48.40 ± 14.5	58 ± 6	0.051
Sex	Male	4	5	7	0.392
	Female	6	5	3	
Reason for extraction	Prosthetic failure	2	4	2	0.303
	Vertical fracture	3	2	5	
	Periodontal disease	3	0	2	
	Caries	2	4	1	
Smoking status	Yes	5	3	1	0.149
	No	5	7	9	
Mean Time of surgery ± S.D. (minutes)		38.50 ± 5	80.00 ± 21	52 ± 16	<0.001

**Table 2:** Healing index at different follow-up times (2 versus 12 weeks) based on different socket preservation protocols. Standard deviation (S.D.).

Healing Index	COLL (Tot. 10)	PC (Tot. 10)	UD (Tot. 10)	One-Way ANOVA *p*-Value
**Mean ± S.D. at 2 weeks**	1.90 ± 1	1.90 ± 0.3	2.00 ± 1.2	0.963
**Mean ± S.D. at 12 weeks**	4.6 ± 0.5	3.5 ± 1.2	3.4 ± 0.5	0.006

## Data Availability

Not applicable.
